# ER CALCIUM SIGNALING COORDINATES MITOCHONDRIAL DYNAMICS AND METABOLIC FUNCTION TO PROMOTE LONGEVITY

**DOI:** 10.1093/geroni/igad104.1134

**Published:** 2023-12-21

**Authors:** Kristopher Burkewitz

**Affiliations:** Vanderbilt University, Nashville, Tennessee, United States

## Abstract

Inter-organelle communication is a critical determinant of cellular metabolic homeostasis, and defects in the interactions between organelles are increasingly correlated with age-dependent diseases. Despite these promising links, exploration of how inter-organelle signaling pathways can be targeted to promote healthy organismal aging is a nascent area of research. We have established a foundation in C. elegans to investigate how the communication between endoplasmic reticulum (ER) and mitochondria, two of the most central metabolic hubs in cells, controls the aging process. Combining electron microscopy, live-imaging and biochemical approaches, we provide evidence that C. elegans forms ER-mitochondrial contacts and that through molecular mechanisms conserved to mammals, ER calcium flux potently stimulates mitochondrial bioenergetics. Furthermore, genetic manipulation of the ER inositol triphosphate receptor (InsP3R) reveals that ER calcium signaling promotes mitochondrial homeostasis at multiple other levels, including mitochondrial gene expression and dynamics. Building on this foundation, we now reveal that ER calcium signaling is essential for longevity in contexts of mitochondrial reprogramming. Surprisingly, InsP3R-stimulated mitochondrial bioenergetics do not appear to be the key mechanism of longevity in these contexts, but rather the underappreciated roles of the ER in tuning mitochondrial gene expression and morphology. Our results thus highlight the InsP3R as a potent and central regulator of diverse mitochondrial properties and reveal that the connections between ER and mitochondria are deeper and more complex than current models suggest. Overall, we reveal that inter-organelle communication between ER and mitochondria is an essential mechanism of longevity in paradigms involving mitochondrial reprogramming.

